# Using Natural Language Processing Methods to Build the Hypersexuality in Bipolar Reddit Corpus: Infodemiology Study of Reddit

**DOI:** 10.2196/65632

**Published:** 2025-03-06

**Authors:** Daisy Harvey, Paul Rayson, Fiona Lobban, Jasper Palmier-Claus, Clare Dolman, Anne Chataigné, Steven Jones

**Affiliations:** 1 Spectrum Centre for Mental Health Research Division of Health Research Lancaster University Lancaster United Kingdom; 2 School of Computing and Communications Lancaster University Lancaster United Kingdom; 3 Lancashire & South Cumbria NHS Foundation Trust Lancashire United Kingdom; 4 School of Mental Health & Psychological Sciences Institute of Psychiatry, Psychology & Neuroscience King's College London London United Kingdom; 5 Lived Experience Researcher London United Kingdom

**Keywords:** bipolar, hypersexuality, natural language processing, Linguistic Inquiry and Word Count, LIWC, BERTopic, topic modeling, computational linguistics

## Abstract

**Background:**

Bipolar is a severe mental health condition affecting at least 2% of the global population, with clinical observations suggesting that individuals experiencing elevated mood states, such as mania or hypomania, may have an increased propensity for engaging in risk-taking behaviors, including hypersexuality. Hypersexuality has historically been stigmatized in society and in health care provision, which makes it more difficult for service users to talk about their behaviors. There is a need for greater understanding of hypersexuality to develop better evidence-based treatment, support, and training for health professionals.

**Objective:**

This study aimed to develop and assess effective methodologies for identifying posts on Reddit related to hypersexuality posted by people with a self-reported bipolar diagnosis. Using natural language processing techniques, this research presents a specialized dataset, the Talking About Bipolar on Reddit Corpus (TABoRC). We used various computational tools to filter and categorize posts that mentioned hypersexuality, forming the Hypersexuality in Bipolar Reddit Corpus (HiB-RC). This paper introduces a novel methodology for detecting hypersexuality-related conversations on Reddit and offers both methodological insights and preliminary findings, laying the groundwork for further research in this emerging field.

**Methods:**

A toolbox of computational linguistic methods was used to create the corpora and infer demographic variables for the Redditors in the dataset. The key psychological domains in the corpus were measured using Linguistic Inquiry and Word Count, and a topic model was built using BERTopic to identify salient language clusters. This paper also discusses ethical considerations associated with this type of analysis.

**Results:**

The TABoRC is a corpus of 6,679,485 posts from 5177 Redditors, and the HiB-RC is a corpus totaling 2146 posts from 816 Redditors. The results demonstrate that, between 2012 and 2021, there was a 91.65% average yearly increase in posts in the HiB-RC (SD 119.6%) compared to 48.14% in the TABoRC (SD 51.2%) and an 86.97% average yearly increase in users (SD 93.8%) compared to 27.17% in the TABoRC (SD 38.7%). These statistics suggest that there was an increase in posting activity related to hypersexuality that exceeded the increase in general Reddit use over the same period. Several key psychological domains were identified as significant in the HiB-RC (*P*<.001), including more negative tone, more discussion of sex, and less discussion of wellness compared to the TABoRC. Finally, BERTopic was used to identify 9 key topics from the dataset.

**Conclusions:**

Hypersexuality is an important symptom that is discussed by people with bipolar on Reddit and needs to be systematically recognized as a symptom of this illness. This research demonstrates the utility of a computational linguistic framework and offers a high-level overview of hypersexuality in bipolar, providing empirical evidence that paves the way for a deeper understanding of hypersexuality from a lived experience perspective.

## Introduction

### Background

Bipolar is a severe mental health condition characterized by recurring episodes of high mood and low mood that is thought to affect at least 2% of the global population [1]. Clinical observations suggest that individuals with bipolar face difficulties regulating emotions and impairments to their cognitive processing, which can contribute to an association with high-risk behaviors [2], and research has demonstrated that these behaviors are often associated with a period of elevated mood [3-5]. Most of the existing research in this area has focused on trying to isolate the biological and behavioral mechanisms that drive risky behavior in people living with bipolar [2,6-14], whereas how these behaviors are exhibited in reality has been comparatively underresearched. Existing research presents a preliminary classification system for the types of risk-taking behavior that people living with bipolar may engage in [3], and through this study, we hope to contribute a more nuanced understanding of one facet of risk-taking behavior, the presentation of hypersexuality, based on large-scale social media data.

This research approaches hypersexuality through the lens of risk-taking behavior and as a symptom of bipolar, focusing on its potential to harm personal safety. However, hypersexuality is a complex concept lacking a universal definition and is shaped by cultural, individual, and situational factors. Perrotta [15] describes it as “a psychological and behavioural alteration as a result of which sexually motivated stimuli are sought in inappropriate ways and often experienced in a way that is not completely satisfactory” and further highlights that hypersexuality is challenging to diagnose due to the lack of established criteria and the impracticality of rigid diagnostic standards in addressing the subjective emotional universe of individuals. Walton et al [16] emphasize that diagnosing hypersexuality requires observable symptoms, subjective perceptions, adverse consequences, and distress. While it is included in the *International Classification of Diseases, 11th Revision*, as compulsive sexual behavior, the rejection of hypersexuality as a distinct diagnosis from the *Diagnostic and Statistical Manual of Mental Disorders, Fifth Edition*, underscores ongoing debates about its classification, reflecting concerns about stigmatization and definitional challenges. The term *hypersexuality* may be used by some individuals to articulate personal experiences without negative consequences, and while these self-descriptions may not align with the definition adopted in this paper, they represent meaningful aspects of lived experience.

There are only a limited number of studies that have focused on the topic of hypersexuality and sexual risk taking in bipolar, and the literature on hypersexuality is sparse and not systematically defined [4,17-19]. Krantz et al [5] found that hypomania often precedes risky sexual behavior, with two-thirds of sexually active youth with bipolar engaging in behaviors categorized as above minimal risk and one-third reporting pregnancy, and Mazza et al [19] observed increased sexual interest in women with bipolar type I compared to bipolar type II. Raja and Azzoni [20] noted high awareness of sexually transmitted infection risks but prevalent risky sexual behaviors among individuals with bipolar, schizophrenia, or schizoaffective disorder, and Marengo et al [21,22] found a link between unplanned pregnancies and hypersexuality in manic episodes, also finding higher rates of casual and nonmonogamous sex among women with bipolar, including during euthymia. Krogh et al [4] explored the impact of mood swings on sexuality in bipolar through qualitative interviews, identifying 5 key themes: sexual drive, behavior, thoughts, intimate relationships, and identity. Their results suggest that elevated mood states increased sexual drive and interactions and that mood-related shifts had significant relational impacts. Observing the existing literature critically, a number of studies that have investigated hypersexuality in bipolar are >30 years old [23-25], making it “subject to the biases of sexual and gender norms” of those times [17]. There is also evidence of stigma attached to hypersexuality and the discussion of sexual experiences from health care professionals [26], as well as a lack of qualitative research into the sexual behaviors of people living with bipolar [4,27].

In this paper, we present a toolbox of computational linguistic techniques, including pretrained machine learning models for demographic inference, the extraction of key psychological domains using the 2022 version of Linguistic Inquiry and Word Count (LIWC-22; Pennebaker Conglomerates, Inc) [28], and unsupervised topic modeling using BERTopic [29], to provide an understanding of what kinds of topics are talked about in discussions regarding hypersexuality. This is the first study to use such methods on data that relate to hypersexuality in general and specifically to bipolar and demonstrates the utility of large-scale language analysis in health research. We acknowledge that there are serious ethical implications associated with the collection of such sensitive information but believe that the benefit of improved understanding and awareness that can be obtained using Reddit (Reddit, Inc) posts is of significant value to people who experience the symptom of hypersexuality as part of their diagnosis of bipolar. We provide a comprehensive outline of our ethical considerations, including consultation with lived experience experts, in the Methods section.

This research aimed to form the foundation for future work in the area by developing a dataset of qualitative information, addressing a significant gap in the field, and presenting key themes. The objective was not to provide an exhaustive analysis of all posts in the dataset as this lies beyond the scope of this study. Instead, the focus of this study was on the methodology used to construct the corpus and on foregrounding this topic as a critical area of scientific interest. We hope that this study supports calls for novel research to “address sexual symptomatology in bipolar within the context of current sexual, cultural, and gender norms” [27]. Our research questions are defined in the following section.

### Research Questions

The research questions for this study were as follows:

- Is hypersexuality talked about on Reddit?
  How can we recognize Redditors who post about hypersexuality on Reddit?
  What are these Redditors’ posting behaviors?
- How can computational linguistic methods be used for exploratory analysis of the Hypersexuality in Bipolar Reddit Corpus (HiB-RC)? This includes the following:
  Psychological domains
  Topic modeling

## Methods

### The Talking About Bipolar on Reddit Corpus

#### Application Programming Interface Data Collection

The posts in this dataset were collected using the Pushshift and PRAW application programming interfaces (APIs) in July 2022 through adaptation of existing code [30]. The 2 subreddits related to bipolar with the highest number of followers—r/bipolar and r/BipolarReddit (approximately 300,000 users)—were scraped to include data posted between July 2017 and July 2022. Applying a similar framework to those in the studies by Coppersmith et al [31], Sekulic et al [32], Cohan et al [33], and Jagfeld et al [34], we then used pattern-matching methods on this corpus to detect Redditors who self-reported a clinical diagnosis of bipolar using a framework implemented by Jagfeld et al [34,35]. We adapted this framework to identify self-reported diagnosis patterns from Reddit posts and comments that (1) contained at least one condition term for bipolar, (2) matched at least one inclusion pattern (ie, bipolar diagnosis of any type by a professional), and (3) did not match any exclusion pattern (eg, self-diagnosis).

After identifying posts from Redditors who had self-reported a diagnosis, we then collected the entire posting history for these users across all subreddits using a custom Python script (Python Software Foundation). This script collected the following information for each comment or submission made by a user: (1) post ID, (2) text body, (3) username, (4) subreddit, (5) post title (for main submissions and not for comments), and (6) time stamp.

We note that there are limitations to using self-reported diagnoses as these have not been clinically verified within the dataset.

#### Demographic Inference

##### Overview

To develop a more comprehensive understanding of the Redditors in our dataset, we used a number of methods for demographic inference (age, gender, and location) presented originally in the work by Jagfeld et al [34], Tigunova et al [36], and Harrigian [37]. While we acknowledge that these methods do not necessarily implement state-of-the-art technologies such as large language models, they are to date the only publicly available models for this type of demographic inference within the Reddit domain. Ethical considerations associated with using inference models are presented in the Ethical Considerations section.

##### Age and Gender

First, we manually identified self-reported instances of age and gender using the pattern-matching code provided in the work by Jagfeld et al [34]. These patterns identify self-reported instances of age and gender from submission titles, which are captured between square brackets as is typical notation on Reddit, for example, “I {28f} am posting here for the first time.” Age was calculated using a function that estimates date of birth based on the age provided in the submission title compared to the submission posting date. Labels for gender were assigned using manual extraction for 675 users, and labels for age were assigned using manual extraction for 643 users. We then used pretrained models to determine age and gender for the remaining users in the dataset for whom a self-reported age or gender could not be determined. The pretrained models used for automated age and gender inference were developed by Tigunova et al [36], who presented a hidden attribute model using a convolutional neural network with attention mechanism architecture to develop representations of demographic information based on language use. The models were trained on similar domain data using the posts from >350,000 Redditors included in the RedDust dataset [36]. The reported accuracies for the age and gender algorithms are an area under the receiver operating characteristic curve of 0.88 for age and an area under the receiver operating characteristic curve of 0.91 and accuracy of 0.86 for gender [36]. Using a subset of gold truth labels that were manually extracted from the dataset for age and gender (675 users for gender and 643 users for age), we manually calculated a weighted *F*_1_ accuracy of 0.8 for gender and 0.6 for age for our dataset. The text used as input to the models was preprocessed before being used as input, which involved cleaning the data to remove hyperlinks and non–English-language words and converting the text to the vector representation format expected by the model (adapting the scripts provided by Tigunova et al [36,38]). Both submissions and comments were used as input to the model provided that the content was between 10 and 100 words in length and that users had at least 10 posts that matched these criteria and using only the most recent 100 posts for each Redditor as input. The inference methods for gender that were used in this study were designed only to detect binary genders (man and woman), the implications of which are discussed further in the Discussion section.

##### Geolocation

We used a pretrained model presented by Harrigian [37] to infer location identifiers for each user in the dataset at the country level. This model was trained using the distribution of words, posts per subreddit, and posts per hour of the day for Reddit users. When applying this model to our data, we included only users with >50 posts and up to 250 posts as specified in the documentation for the package to improve the accuracy of predictions [39]. The global model provided by Harrigian [37] was used, which achieves 35.6% accuracy, and as reported by Jagfeld et al [34], the accuracy is generally higher for users with more training data (95.1% for the United States, 65.1% for Canada, 82.8% for the United Kingdom, 44.1% for Australia, and 41.1% for Germany).

#### Developing the HiB-RC

After implementing the inference models, any users whose posting history did not satisfy the criteria for the pretrained models were removed from the dataset. This resulted in a snapshot corpus that contains data that span 13 years, with the earliest post dating back to June 2009 and the latest submission date in August 2022.

To detect posts with content related to hypersexuality, we created an initial set of seed terms to generate a subcorpus (the HiB-RC) of users with a self-reported history of hypersexuality. To develop this vocabulary of seed terms, we identified the keywords and phrases related to hypersexuality from a previous study that used lived experience interview data [3] and trained both word2vec (Google AI) [40] and fastText (Facebook’s Artificial Intelligence Research laboratory) [41] embedding models on the Talking About Bipolar on Reddit Corpus (TABoRC) to find synonyms (words and phrases) and misspellings of these keywords and phrases. The fastText algorithm produces character-level embeddings that find numeric representations of words by looking at their character-level compositions, thus enabling us to detect common typographical errors for the hypersexuality seed terms. Traditional word- and character-level embeddings were deemed to be sufficient for this task as the embeddings were not being used as part of a predictive algorithm and, thus, there was a cost benefit in terms of lower computational and environmental cost for training these simpler models versus fine-tuning a contextual large language model. The final list of seed terms used to collect posts related to hypersexuality is presented in Textbox 1.

**Hypersexuality keywords used to create the Hypersexuality in Bipolar Reddit Corpus. These keywords were generated by finding the most similar terms to the input keywords using word2vec (Google AI) and fastText (Facebook’s Artificial Intelligence Research laboratory) embeddings trained on the Talking About Bipolar on Reddit Corpus.**

**Input keyword to the word2vec and fastText models**

- “Hypersexual”
- “Hypersexuality”
- “Hyper-sexual”
- “Hyper_sexual”

**Output—*most similar* keywords**

- “Hypersexual”
- “Hyper sexual”
- “Hypersexuallity”
- “Hypersex”
- “Hyper sexualised”
- “Hyper sexuality”
- “Oversexual”
- “Hyposexual”
- “Hyper sexualized”
- “Hypersexualized”
- “Overly sexual”
- “Hyper sexualization”
- “Hypersexualization”
- “Hyposexuality”
- “Hypersexuality”

At the early stages of data collection, we used a much longer list of seed terms to search for posts related to hypersexuality, including phrases such as “hook up with strangers,” “high sex drive,” and “threesomes.” This list of vocabulary was generated using the same word embedding methodology but included a more diverse set of keywords as input when using the models to search for similar words and phrases. This resulted in a much noisier dataset where it was apparent after manual inspection that a large number of the posts were not written in the context of experiencing hypersexuality as a symptom but rather in the context of people sharing and discussing sexual experiences. Due to the infancy of this field of work and to avoid compounding the stigma regarding sex or incorrectly categorizing diverse sexual experiences as hypersexuality, we chose to refine the keyword list used as input to the word embedding models to words and phrases that directly related to the notion of “hypersexuality.” We considered it more ethical to collect data from instances in which individuals self-reported the symptom of hypersexuality rather than inferring hypersexuality through more nuanced descriptions of sexual behavior. The result was that there was less ambiguity and greater reliability in the dataset of posts, with the disadvantage that we filtered out an unknown amount of data related to hypersexuality that talked about the topic in more nuanced ways. We refer in this paper to the concept of a corpus being “acceptably representative,” whereby “we have to make do with studying merely a sample of the language use, or variety, as a whole” due to restrictions on time and resources and, in this case, ethical considerations [42].

After we had generated the final seed list of hypersexuality terms, we created a filter and applied this to the TABoRC. After preprocessing the returned posts to remove duplicates and only include posts that were >30 words in length, we manually annotated this dataset using the doccano tool to verify a post’s inclusion in the corpus, with the posts annotated as confirming a hypersexuality report forming the HiB-RC. The corpus was annotated in full by DH, and circa 10% of the corpus (300 posts) was annotated by second and third annotators (SJ and PR). Interannotator agreement achieved a Krippendorff α score of 0.77 [43], and majority voting was used to solve annotator disagreements. Disagreements primarily occurred in cases in which an experience of hypersexuality was described but there was ambiguity on whether the author of the post was the one who had experienced the symptom. The annotation guidelines are presented in Multimedia Appendix 1.

### Analysis Methodology

#### Interpreting the HiB-RC

To begin the exploratory analysis of our dataset, we produced descriptive statistics to detail the user and posting characteristics of the corpus. These analyses were conducted using Python, and the results are presented in the Results section to show demographic characteristics, the number of new users posting in the HiB-RC each year and the number of new posts referencing hypersexuality each year (using the TABoRC as a comparison dataset), and the top subreddits to which posts about hypersexuality were posted.

#### Linguistic Inquiry and Word Count

After exploring the Redditor characteristics of our dataset, we used LIWC-22 [28] to understand the key psychological domains within the HiB-RC.

LIWC-22 is a text analysis application that maps psychosocial constructs to words, phrases, and linguistic constructions [28]. Linguistic Inquiry and Word Count (LIWC) processes text using software and a dictionary, where the dictionary contains groups of words that relate to a particular domain (eg, positive or negative tone). Documents of interest (the input text) are analyzed by the software to map the domains to the text, calculating the percentage of each document that comprises words in these dictionary domains. LIWC was designed on the premise that the words that people use tell us about “their psychological states: their beliefs, emotions, thinking habits, lived experiences, social relationships, and personalities” [28]. The LIWC-22 dictionary is based on >12,000 words, phrases, and emoticons, and the authors describe that “in the advent of more powerful analytic methods and more diverse language samples, we have been able to build more internally consistent language dictionaries with enhanced psychometric properties” in this latest release of the software [28]. Modern text analysis has been influenced by >100 years of psychological research [44], and previous research has demonstrated how language analysis can provide insights into cognitive mechanisms, with “an increasing number of studies [which] demonstrate, [that] the ways in which people use words is reliable over time” [45].

LIWC domains have been used in various existing studies that explore how language is used by people living with bipolar, including as input for prediction and classification models [31-33,46-53] and exploratory analysis of mental health datasets [54,55]. In this research, we used LIWC to identify psychological domains that appear significantly more or less by comparing the HiB-RC to a control corpus formed of the same users’ entire posting history across Reddit.

#### Modeling Hypersexuality

Egger and Yu [56] describe that social media data have opened up new pathways for scientific research but that the short and unstructured nature of the documents within social media datasets can cause methodological issues for analysis. The authors describe that topic modeling has increasingly been applied to the topic of social science, where topic models are defined as “probabilistic models for uncovering the underlying semantic structure of a document collection” [57].

Topic models seek to identify patterns between similar documents to add structure to an otherwise unstructured collection of text to facilitate exploration and understanding. Latent Dirichlet allocation (LDA) is one of the most widely used traditional methods for topic modeling and is a generative statistical model introduced by Blei et al [58]. Despite the popularity of LDA, the reliability and validity of the results have been criticized because there is no definitive method of model evaluation and there is a lack of guidance related to fine-tuning. The efficacy of LDA for analyzing social media data has been further criticized because the noisy and sparse datasets generated in social science research often do not contain enough features for statistical learning [56].

More recent topic-modeling algorithms that have been implemented as an alternative to LDA [56] include embedding models [29,59] that rely on the vectorization of text data to locate semantically similar words and documents. BERTopic [29] is an algorithm that uses pretrained embedding models to create word and document embeddings so that documents that occupy similar vector space can be grouped together to form topics. By default, BERTopic incorporates Bidirectional Encoder Representations From Transformers embeddings and a term frequency–inverse document frequency algorithm, which compares the importance of terms within a cluster and creates term representation based on this [60]. This means that the higher the value is for a term, the more representative it is of its topic. Due to the sparse nature of social media data, BERTopic also includes a default module for dimension reduction using uniform manifold approximation and projection, which enables these dimensions to be reduced to the extent that hierarchical density-based spatial clustering of applications with noise can be used to identify dense regions in the documents [56,59].

On the basis of the comparison of topic-modeling methods presented in the work by Egger and Yu [56], BERTopic presents a number of advantages that influenced our decision to use this method in our research. These include its ability to perform well across multiple domains due to the use of pretrained embeddings and the fact that little to no preprocessing of text is required before training. There still remain limitations, which are described in the Discussion section.

### BERTopic Setup

BERTopic was adapted for this study from the code provided by Grootendorst [61]. The parameters that had a significant impact on the topic output included the following:

First, KeyBERTInspired as the main representation input to the model. KeyBERTInspired [62] extracts representative keywords for topics using word embeddings, ensuring more context-aware representations. First, document embeddings are generated to capture the overall meaning of a document. Word embeddings are then created for N-gram words and phrases. Finally, cosine similarity is used to identify the words and phrases that are most similar to the document embedding. Textbox 2 shows the difference in representations produced using the default term frequency–inverse document frequency and KeyBERTInspired representation models.

Second, the use of *mxbai-embed-large-v1* sentence embeddings [63] as the pretrained embeddings for the model, which demonstrate very high performance for low memory use (ranked 13 in the Massive Text Embedding Benchmark leaderboard at the time of writing). We also tested topic generation using MentalBERT embeddings that have been trained on Reddit data within the mental health domain, but the resulting topic representations were less defined and noisier [64].

Third, a custom list of stop words were provided to the CountVectorizer module and, thus, excluded from clusters after training. This list included generic English stop words (eg, “and,” “or,” “this,” and “was”) as well as frequently occurring words such as “hypersex*” and “bipolar”—keywords that appeared in nearly every post due to the seed list of vocabulary used to generate the corpus or the topic domain.

**Default versus KeyBERTInspired representation of the example topics generated by BERTopic.**

**Default representation**

- “Ve,” “manic,” “feel,” “really,” “don,” “mania,” “time,” “people,” “sleep,” and “know”
- “Age,” “years,” “sexual,” “older,” “csa,” “remember,” “trauma,” “know,” “young,” and “happened”

**KeyBERTInspired representation**

- “Hypomanic,” “manic,” “mania,” “depressed,” “depressive,” “depression,” “disorder,” “psychiatrist,” and “mood”
- “Abuser,” “abused,” “abuse,” “sexual,” “trauma,” “memories,” “rape,” “therapy,” “touched,” and “older”

After our model setup had been finalized, we manually merged similar topics after inspecting the posts included within each topic using the *merge_topic()* method of the model. Finally, we manually assigned topic labels for our topics to be used in visualizations and saved the model as a pickle file for future analysis. As noted when describing the limitations of BERTopic, the topics produced by the model may change each time the model is run. After altering the parameters of the model, implementing *mxbai-embed-large-v1* as the sentence embedding model, and using KeyBERTInspired as the main representation model, we found the generation of topics to be relatively stable with each iteration.

### Ethical Considerations

We recognize the importance of developing an ethical framework when working with sensitive data that describe personal lived experience, especially when collecting data from a public site such as Reddit. We outline in this section our considerations regarding consent, anonymization, the right to be forgotten, and dataset retention. Our framework was informed by multiple sources, including institutional resources from the British Psychological Society, the British Sociological Association, and the UK government [65-67] as well as sources from academic research and guidelines [34,68-72]. This study was conducted as part of a PhD thesis on the topic of risk-taking behaviors in bipolar, and we consulted a panel of lived experience advisors through Lancaster University Spectrum Connect at the early stages of design. We also engaged with Bipolar UK on a webinar on hypersexuality in 2024 [73] and sought invaluable guidance from lived experience researchers who coauthored this paper. Ethics approval was granted for the project by Lancaster University in December 2021 (FHMREC21042).

Reddit is colloquially known as “the front page of the internet,” with >50 million daily users and 100,000 *active* subreddits in 2024 [74,75], and research has shown that the anonymity afforded by social media sites enables users to self-disclose on sensitive topics that they may otherwise find difficult to talk about [76]. As researchers, we wholly acknowledge that the Reddit posts used in our study contain sensitive information and that the forum users were not aware that their discussions would be used for research. We did not seek informed consent from the Redditors whose posts we collected due to the impractical nature of this task considering that the posts of >5000 Redditors were included in the TABoRC, but we note that Reddit users are made aware that their posts are publicly accessible through Reddit’s terms and conditions. From a legal perspective, although Reddit is by nature an anonymous platform, we cannot know that Redditors do not use the same username across other social media sites or platforms, and therefore, we treat the information collected from the site as personal data. In accordance with the Data Protection Act 2018 and General Data Protection Regulation, an exemption for conducting research for “special purposes” would be relevant for nonconsent as we intend to publish our research and are confident that the publication of any research associated with the collection of these Reddit data “would be in the public interest” [67]. Further to the legal grounding of work conducted in the public interest, the motivation for this study was to learn more about experiences of a typically stigmatized symptom to identify how people experiencing hypersexuality could be better supported. There is existing evidence from lived experience suggesting that data on this topic can be difficult to access within a health care setting, so we acknowledge the limitations of using data sourced from the web but also recognize the unique insights that the analysis of such data can provide [3,27,77].

Following previous guidance [65,68,69], as we did not rely on consent for this study, we masked the usernames in this dataset (created alternative alphanumeric usernames for each Redditor in the dataset) and have only included paraphrased and depersonalized quotes in research outputs. We have also minimized the amount of qualitative data reported by using computational methods such as topic modeling and LIWC, which enable us to present key themes and insights from the data in an aggregate format without needing to rely heavily on quotes. Where we presented paraphrased quotes, we verified that Redditors could not be reidentified based on an internet search of the reworded quotes. Using these methods, we strived to maintain the privacy of the Redditors included in our corpus as much as possible.

We would also like to draw attention to the demographic inference methods that we used. Performing inference of such data enables us to offer predicted demographic information about the study population, which may allow for comparison to other domains, for example, clinical populations. Reporting on aspects such as gender also contributes toward more ethical natural language processing data collection as these predictions can suggest how experimental results might be generalized and also highlights where the data include bias [78]. However, inferring demographic information adds an extra level of personal data to the corpus, and we acknowledge that this comes with its own risks. The demographic data that we inferred are not intended to be used for identification or targeting of users in any way, and we understand that these inferred statistics are not 100% accurate, nor have they been used as features in any predictive models. The demographic data were only reported in aggregate format and will not be publicly released, although the code used is available open source. We would also like to strongly emphasize that any analysis reported using the demographic data indicates correlation and not causality.

Using Reddit as a primary data source is not “wholly problematic or must be ceased,” but “careful handling and anonymization of such materials is of paramount importance for maximising ethical research practice going forward” [71]. We have decided to only publish redacted versions of both the TABoRC and HiB-RC with the UK Data Service, as requested by the funder of this research (the Economic and Social Research Council). The redacted versions of the datasets will include only the IDs for the posts that form the corpora. The corpora will be disseminated upon request on a case-by-case basis to researchers with an institutional email address, and future researchers will be required to access the content of the posts using an API. This complies with Article 17 of the UK General Data Protection Regulation and an individual’s rights to data erasure because any content that has been removed since the creation of our datasets will appear as “[removed]” upon retrieving the post ID using an API.

## Results

### Posting Characteristics on Reddit

The TABoRC comprises 6,679,485 posts from 5177 users, and the HiB-RC comprises 2146 posts from 816 users. The demographic statistics for the TABoRC and HiB-RC corpora are presented in Table 1. The data suggest that >15% (816/5177, 15.76%) of the users in the TABoRC reported experiences of hypersexuality.

**Table 1:** Demographic information for the Hypersexuality in Bipolar Reddit Corpus (HiB-RC), the Talking About Bipolar on Reddit Corpus (TABoRC), and the benchmarking dataset [34].

				Proportion of users
**TABoRC (n=5177), n (%)**				
	**Age (y)^a^**			
		14-23 (teenagers and young adults)	1385 (26.8)	
		24-45 (adults)	3371 (65.1)	
		46-65 (middle-aged adults)	389 (7.5)	
		66-100 (older adults)	32 (0.6)	
	**Gender**			
		Female	3668 (70.8)	
		Male	1509 (29.1)	
	**Country**			
		United States	3970 (76.7)	
		United Kingdom	366 (7.1)	
		Canada	337 (6.5)	
		Germany	108 (2.1)	
		Australia	100 (1.9)	
		Sweden	58 (1.1)	
		Other countries	238 (4.6)	
**HiB-RC (n=816),** **n (%)**				
	**Age (y)^a^**			
		14-23 (teenagers and young adults)	207 (25.4)	
		24-45 (adults)	531 (65.1)	
		46-65 (middle-aged adults)	74 (9.1)	
		66-100 (older adults)	4 (0.5)	
	**Gender**			
		Female	626 (76.7)	
		Male	190 (23.3)	
	**Country**			
		United States	600 (73.5)	
		United Kingdom	62 (7.6)	
		Canada	61 (7.5)	
		Germany	21 (2.6)	
		Australia	24 (2.9)	
		Sweden	12 (1.5)	
		Other countries	36 (4.4)	
**Benchmarking dataset [1]^b^, %**				
	**Mean age (y)**			
		13-17	16.1	
		18-29	29.8	
		30-49	47.5	
		50-64	6.6	
		≥65	0	
	**Gender**			
		Female	52.2	
		Male	47.8	
	**Country**			
		United States	81.9	
		United Kingdom	5.6	
		Canada	4.9	
		Germany	1.4	
		Australia	1.7	
		Sweden	—^c^	
		Other countries	4.5	

^a^The pretrained model [2] included an additional age category of 0 to 13 years (child). For any users who were manually or automatically included within this age group, we removed their data from the dataset as Reddit requires a minimum sign-up age of 13 years.

^b^Original data values were not provided with the dataset, so we have only presented percentages in this section.

^c^Not available.

**Figure 1:**
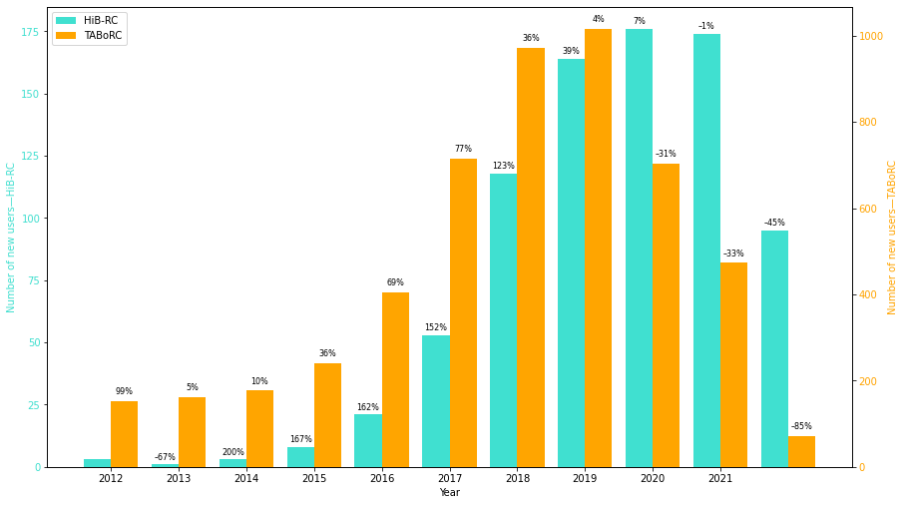
Comparing the number of new users each year in the Hypersexuality in Bipolar Reddit Corpus (HiB-RC) and the Talking About Bipolar on Reddit Corpus (TABoRC). There is no percentage increase reported for the HiB-RC in 2012 because the first post in the HiB-RC was reported in 2012. Data collection ended in July 2022, so the observed trend in user growth may not fully reflect subsequent changes.

**Figure 2:**
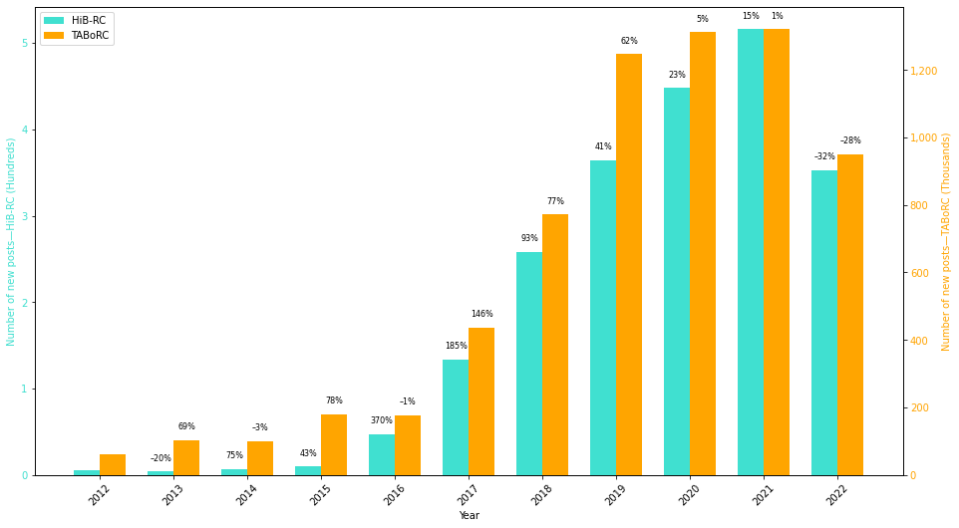
Comparing the number of new posts each year in the Hypersexuality in Bipolar Reddit Corpus (HiB-RC) and the Talking About Bipolar on Reddit Corpus (TABoRC). There is no percentage increase reported for the HiB-RC in 2012 because the first post in the HiB-RC was reported in 2012. Data collection ended in July 2022, so the observed trend in post growth may not fully reflect subsequent changes.

Figure 1 compares the number of new users between 2012 and 2021 in the HiB-RC and the TABoRC, with an average yearly increase of 86.97% (SD 93.8%) and 27.17% (SD 38.7%), respectively. Figure 2 compares the number of new posts between 2012 and 2021 in the HiB-RC and the TABoRC, with an average yearly increase of 91.65% (SD 119.6%) and 48.14% (SD 51.2%), respectively. The bars represent the raw number of posts and the labels demonstrate the yearly percentage increase compared to the previous year. Table 2 shows how many posts that reference hypersexuality are made by each user.

Table 3 shows the top subreddits where posts related to hypersexuality were made within the HiB-RC (where >5 posts were made to the same subreddit), with the most visited subreddits including r/bipolar, r/BipolarReddit, r/bipolar2, r/AskReddit, and r/BipolarSOs.

**Table 2:** Number of posts per user referencing hypersexuality (N=816).

Number of posts per user referencing hypersexuality	Users, n (%)
1	453 (55.5)
≥1 to <5	270 (33.1)
≥5 to ≤10	65 (8)
>10	28 (3.4)

**Table 3:** Top subreddits for posts related to hypersexuality (where >5 posts were made to the same subreddit; N=2146).

Subreddit	Posts, n (%)
r/bipolar	1027 (47.86)
r/BipolarReddit	421 (19.62)
r/bipolar2	169 (7.88)
r/AskReddit	53 (2.47)
r/BipolarSOs	43 (2)
r/polyamory	28 (1.3)
r/BPD	28 (1.3)
r/hypersexuality	26 (1.21)
r/sex	16 (0.75)
r/adultsurvivors	13 (0.61)
r/ADHD	11 (0.51)
r/BDSMAdvice	10 (0.47)
r/CPTSD	10 (0.47)
r/relationship_advice	9 (0.42)
r/AskRedditAfterDark	9 (0.42)
r/demisexuality	8 (0.37)
r/relationships	7 (0.33)
r/AskMen	6 (0.28)
r/BorderlinePDisorder	6 (0.28)
r/depression	6 (0.28)
r/mentalillness	6 (0.28)

### LIWC Results

Table 4 presents a selection of LIWC domains that were statistically significant when comparing the HiB-RC to a control corpus from the same users. The control corpus contains all posting history from each user in the HiB-RC across Reddit after removing the posts that are included in the HiB-RC. The total word count of the HiB-RC is 344,786, and the total word count of the control corpus is 69,495,570. We built the control corpus based on the hypothesis that these data would be representative of more general language use across Reddit by the same group of users based on manual inspection of a sample of the data. After identifying a nonnormal distribution in most LIWC domains based on paired scores using the Shapiro-Wilk test [79], we determined statistical significance using a paired Wilcoxon signed rank test [80] to identify significant differences in domain scores between the control and hypersexuality corpora. All domains included in Table 4 are significant at a *P* value of <.001. The table presents the Wilcoxon score and associated *P* value together with the effect size (Cohen *d*, with directionality represented by the minus sign [–]), which ranges between small (0.01 to 0.2) and huge (≥2) [81]. The methodology for the LIWC analysis was adapted from the work by Cohan et al [33].

**Table 4:** Significant Linguistic Inquiry and Word Count domains in the Hypersexuality in Bipolar Reddit Corpus (HiB-RC) compared to a control corpus of Reddit posts from the same set of users.

Domain		Description or most frequently used exemplars (from LIWC-22^a^ dictionary)	Direction of significance^b^	Wilcoxon signed rank score	*P* value	Cohen *d*
**Linguistic dimensions**						
	First person singular	“I,” “me,” “my,” and “myself”	Positive	34,402.0	<.001	0.37
	First person plural	“We,” “our,” “us,” and “lets”	Negative	66,744.0	<.001	−1.14
	Second person	“You,” “your,” “u,” and “yourself”	Negative	52,244.5	<.001	−0.55
	Third person singular	“He,” “she,” “her,” and “his”	Negative	71,742.5	<.001	−0.55
	Third person plural	“They,” “their,” “them,” and “themsel*”	Negative	49,597.5	<.001	−1.57
**Psychological processes**						
	Achievement	“Work,” “better,” “best,” and “working”	Negative	91,466.0	<.001	−0.61
	Power	“Own,” “order,” “allow,” and “power”	Negative	111,908.5	<.001	−0.34
	Cognition	“Is,” “was,” “but,” and “are	Positive	126,646.5	<.001	0.09
	Cognitive processes	“But,” “not,” “if,” “or,” and “know”	Positive	126,921.0	<.001	0.09
	Insight	“Know,” “how,” “think,” and “feel”	Positive	138,386.0	<.001	0.17
	Positive tone	“Good,” “well,” “new,” and “love”	Negative	95,852.5	<.001	−0.36
	Negative tone	“Bad,” “wrong,” “too much,” and “hate”	Positive	119,137.5	<.001	0.27
	Emotion	“Good,” “love,” “happy,” and “hope”	Positive	132,424.5	<.001	0.24
	Positive emotion	“Good,” “love,” “happy,” and “hope”	Negative	131,386.0	<.001	−0.12
	Negative emotion	“Bad,” “hate,” “hurt,” and “tired”	Positive	30,310.0	<.001	0.34
	Social behavior	“Said,” “love,” “say,” and “care”	Negative	121,529.5	<.001	−0.16
	Prosocial behavior	“Care,” “help,” “thank,” and “please”	Negative	107,645.5	<.001	−0.22
	Politeness	“Thank,” “please,” “thanks,” and “good morning”	Negative	64,811.0	<.001	−1.63
	Communication	“Said,” “say,” “tell,” and “thank*”	Negative	105,069.0	<.001	−0.42
	Social referents	“You,” “we,” “he,” and “she”	Negative	46,417.5	<.001	−0.39
	Family	“Parent*,” “mother*,” “father*,” and “baby”	Negative	98,628.5	<.001	−0.31
	Female references	“She,” “her,” “girl,” and “woman”	Negative	84,008.5	<.001	−0.37
	Male references	“He,” “his,” “him,” and “man”	Negative	96,669.5	<.001	−0.29
**Expanded LIWC-22 dictionary**						
	Lifestyle	“Work,” “home,” “school,” and “working”	Negative	53,011.0	<.001	−0.69
	Leisure	“Game*,” “fun,” “play,” and “party*”	Negative	82,334.0	<.001	−0.74
	Home	“Home,” “house,” “room,” and “bed”	Negative	66,942.5	<.001	−1.52
	Work	“Work,” “school,” “working,” and “class”	Negative	57,181.0	<.001	−0.96
	Money	“Business*,” “pay*,” “price*,” and “market*”	Negative	94,900.5	<.001	−0.51
	Religion	“God,” “hell,” “christmas*,” and “church”	Negative	78,149.5	<.001	−0.47
	Physical	“Medic*,” “food*,” “patients,” and “eye*”	Positive	64,808.5	<.001	0.38
	Health	“Medic*,” “patients,” “physician*,” and “health”	Positive	97,079.0	<.001	0.31
	Wellness	“Healthy,” “gym*,” “supported,” and “diet”	Negative	50,662.5	<.001	−2.35
	Mental health	“Mental health,” “depressed,” “suicid*,” and “trauma*”	Positive	73,266.5	<.001	0.58
	Substances	“Beer*,” “wine,” “drunk,” and “cigar*”	Negative	73,783.0	<.001	−0.29
	Sexual	“Sex,” “gay,” “pregnan*,” and “dick”	Positive	40,559.5	<.001	0.78
	Reward	“Opportun*,” “win,” “gain*,” and “benefit*”	Negative	52,059.0	<.001	−2.45
	Time	“When,” “now,” “then,” and “day”	Positive	106,340.5	<.001	0.22
	Past focus	“Was,” “had,” “were,” and “been”	Positive	125,182.5	<.001	0.14
	Future focus	“Will,” “going to,” “have to,” and “may”	Negative	72,929.0	<.001	−0.90

^a^LIWC-22: 2022 version of Linguistic Inquiry and Word Count

^b^Positive direction indicates that the domain is more prevalent in the HiB-RC than the control corpus. Negative direction indicates that the domain is less prevalent in the HiB-RC than the control corpus.

### BERTopic Results

Our implementation of BERTopic initially yielded 14 topics and 1 outlier class (which contained posts that were determined to be too noisy to accurately cluster into one of the topics by the algorithm). After manual analysis of these topics, we merged a number of similar clusters using the inbuilt function in BERTopic to produce 9 final topics (shown in Table 5).

**Figure 3:**
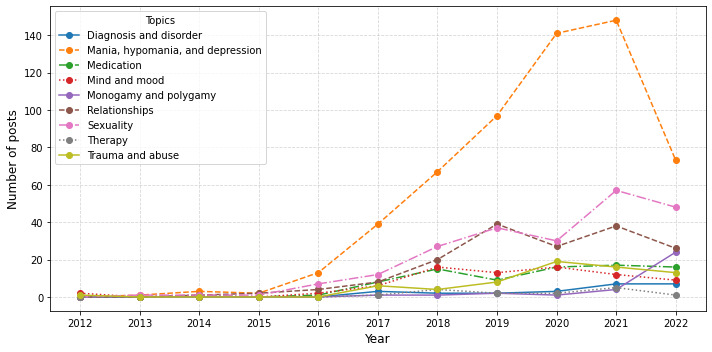
Graph representing the dynamic topic modeling over time. Data collection ended in July 2022, so the observed trends may not fully reflect subsequent changes.

Figure 3 shows how the representation of hypersexuality topics has changed over time, with all topics showing an increase in representation since the inception of the dataset.

**Table 5:** Topics produced by BERTopic (with the manually inferred topic name), the top 10 keywords for each cluster, and paraphrased excerpts from the most representative post for each topic. Additional examples for each topic are provided in Multimedia Appendix 1 (n=2146).

Topic name (inferred)	Posts, n (%)	Top 10 keywords in the cluster	Extract from the most representative post for each topic (paraphrased)
—^a^	878 (40.91)	Outliers	—
Mania, hypomania, and depression	584 (27.21)	“Hypomanic,” “hypomania,” “manic,” “mania,” “disorder,” “depressive,” “depressed,” “depression,” “diagnosed,” and “psychiatrist”	“Over 3-4 months, I left home, almost divorced, and indulged in reckless sexual encounters due to hypersexuality, hurting my family and behaving poorly. Reflecting on my manic episode, I now see the embarrassment and realize it’s a common experience for many. As I came down, I recognized my strange behavior.”
Sexuality	221 (10.3)	“Sexuality,” “sexually,” “sexual,” “relationship,” “feelings,” “manic,” “bisexual,” “aroused,” “feeling,” and “boyfriend”	“I define myself as demisexual because I only experience attraction towards those I’m emotionally connected to, none of whom share the sentiment. Despite this, I have a strong sexual drive, feeling intense arousal monthly, and occasionally endure extended periods of hypersexuality lasting days or weeks.”
Relationships	165 (7.69)	“Relationship,” “relationships,” “manic,” “boyfriend,” “disorder,” “sexuality,” “mania,” “dating,” “mental,” and “diagnosed”	“I’m a challenging partner due to my manic episodes, leading to outbursts, bouts of hypersexuality (increasing the temptation to cheat), excessive drinking, and impulsive life-altering choices. Also, I believe I haven’t completely healed from my previous abusive relationship.”
Medication	83 (3.87)	“Hypomanic,” “hypomania,” “lamictal,” “manic,” “wellbutrin,” “seroquel,” “antipsychotic,” “lithium,” “zoloft,” and “psychiatrist”	“In the last two months of taking it, there’s been no improvement. Even after a week on 200mg, I’m still stuck in a severe mixed episode. I’m overwhelmed with hypersexuality, impulsivity, late nights, and a complete lack of motivation. My mood appears to be cycling rapidly, possibly even faster than before.”
Mind and mood	76 (3.54)	“Hypomanic,” “manic,” “mood,” “mania,” “lithium,” “feeling,” “anxiety,” “days,” “thoughts,” and “mind”	“I’m beginning to understand that although I experience cycling, my episodes often extend beyond a few days. Recent weeks of mood tracking reveal durations of a week or even two, with my current mood episode already lasting four days. In this most recent episode I’ve been feeling hypersexual, and like my head is full of thoughts. I’m also anxious and I’ve been focusing a lot on work.”
Trauma and abuse	67 (3.12)	“Abuser,” “abused,” “abuse,” “sexual,” “raped,” “trauma,” “feelings,” “memories,” “therapy,” and “touched”	“I started having cyber-sex with men in their 20s when I was 13, I would have online sex with anyone who was there, I wasn’t thinking about their age. After this hypersexuality, I became very anxious and scared of men, and now I become very triggered when the topic of sexual abuse comes up.”
Monogamy and polygamy	33 (1.54)	“Polyamory,” “polyamorous,” “monogamy,” “monogamous,” “relationship,” “relationships,” “poly,” “married,” “spouse,” and “boyfriend”	“Following almost two decades of monogamous marriage, I divorced due to manic hypersexuality from bipolar, finding monogamy challenging. For five years, I explored different non-monogamous arrangements, aiming to find a new partner for monogamy. However, after another failed attempt, I encountered a married polyamorous man and chose to explore that avenue instead.”
Diagnosis and disorder	24 (1.12)	“Disorder,” “sexually,” “sexual,” “addiction,” “manic,” “adolescence,” “mania,” “psychological,” “addicts,” and “diagnosed”	“At 32, I was diagnosed with BP2, prompting reflection on missed signs in my childhood and adolescence. Back then, mental health wasn’t a focus in my large family, and I concealed much of my struggles. With a BPD diagnosis too, distinguishing between disorders complicates understanding my experiences and symptoms. I completely relate to the hypersexuality. I have been very sexual since my early teens with a boyfriend who was years older than me.”
Therapy	15 (0.7)	“Therapist,” “therapy,” “therapists,” “counseling,” “psychologist,” “relationship,” “intimacy,” “psych,” “helped,” and “talking”	“I always remember them saying to never underestimate libido although that may not be the best advice for someone who’s hypersexual.”

^a^This is the outlier category that is automatically created by BERTopic to filter posts that are ambiguous and cannot be clustered into one of the topics.

## Discussion

### Posting Behaviors

The results demonstrate that natural language processing methods were successfully used to create a corpus of Reddit posts from users who had self-reported a diagnosis of bipolar and who created content that relates to hypersexuality. There were 816 users in the dataset who posted to Reddit about hypersexuality, forming a corpus of >2000 posts. While most of the users (453/816, 55.5%) in the HiB-RC had only posted about hypersexuality once (within the data that we collected), 44.5% (363/816) of the Redditors did post repeatedly about hypersexuality—which could indicate repeat episodes of hypersexuality or sharing the same experience across multiple threads. The data demonstrate that there has been a substantial increase in the discussion of hypersexuality in terms of both the number of posts and the number of users when comparing the HiB-RC posts to the TABoRC, suggesting that this is a salient topic being discussed on Reddit.

The data suggest that the HiB-RC encompasses approximately 15% of the Redditors from the TABoRC (816/5177, 15.76%), although the number of Reddit users who talk about hypersexuality more widely on Reddit is likely to be much higher than this. We make this assumption based on the fact that we used a restrictive set of keywords and phrases to retrieve posts related to hypersexuality, as discussed in the Methods section, and based on reports that 63% of women in a recent survey on experiences of bipolar reported hypersexuality as a symptom of bipolar [27,82]. Our dataset relied on Redditors who had self-reported a diagnosis and were already aware of the terminology of “hypersexuality,” but we recognize that there is a large number of people who may be sharing their hypersexual experiences on the web before receiving a diagnosis using nonclinical terminology without knowing that this is a symptom of bipolar [27,77,82]. This is an important area of exploration for future research.

When comparing the demographic inference of the HiB-RC to data from a study that profiled Reddit users with a self-reported diagnosis of bipolar [34], our statistics for age and geolocation correlate. Most Redditors in the HiB-RC were based in the United States, the United Kingdom, Canada, Germany, and Australia (768/816, 94.1%) and were between the ages of 24 and 45 years (531/816, 65.1%). However, the inferred gender data for the TABoRC suggest that most Redditors were women (3668/5177, 70.85%), which is an interesting observation compared to findings that most Reddit users in general are men [83] and previous research on bipolar that identified a more equitable distribution of Redditors who present as men and women [34]. One interpretation could stem from different methodologies of data collection; we initially sourced our Redditors from subreddits that were specific to bipolar, whereas Jagfeld et al [34] sourced Redditors across Reddit from the outset. This notion correlates with research that Redditors who present as women are 33% more likely to post in mental health–related subreddits than Redditors who present as men [55] and, thus, we would assume are also more likely to self-report a diagnosis of bipolar in these subreddits. This gender inequality is further conflated in the HiB-RC (626/816, 76.7% of the dataset presented as women). While the interpretation of this statistic requires consideration of a number of sociological perspectives and a full understanding of this topic is beyond the scope of this study, existing research reports on the “sexual double standard” [84,85]. It is well documented that “behaviours associated with high sexual activity [are] expected more and evaluated more positively” [84] in men than in women, and therefore, it is conceivable that women could feel more stigmatized about hypersexual experiences and may be more likely to post in an online “safer” space [76]: “women must strike the right balance between what society deems to be too much sex or not enough; men suffer from the pressure of performance” [77].

Finally, when considering where Redditors in the HiB-RC posted, we can observe that 77.82% (1670/2146) of the content was posted in subreddits associated with bipolar (r/bipolar, r/BipolarReddit, r/bipolar2, and r/BipolarSOs), suggesting that most of the Redditors in the dataset were aware that this is a symptom that is linked to bipolar. As described previously, this corpus is unlikely to be fully representative of the multiple and nuanced ways in which hypersexuality could be described on the web, and therefore, we should not misrepresent this statistic and assume that the wider population of people with a diagnosis of bipolar are aware of hypersexuality as a symptom. We also note that 7.88% (169/2146) of the posts appeared in the r/bipolar2 subreddit, which has typically been ignored in academic literature related to hypersexuality in bipolar [27,86].

### LIWC Analysis

The significant LIWC domains presented in the HiB-RC yielded a number of interesting insights, of which we will only discuss the most salient in this section.

With reference to the *cognition* domains, posts in the HiB-RC were more likely to demonstrate *negative tone* and *negative emotion* and less likely to present *positive tone* and *positive emotion*. This is logical when we consider the potential impact that the symptom of hypersexuality can have on a person’s life and correlates with the significantly higher presence of the *mental health* domain, which matches words such as *depressed*, *suicide*, and *trauma*. It is also logical that the *sexual* domain was significantly more frequent in the HiB-RC, where Redditors focused on sharing sexual experiences. For the domains of *reward* and *wellness*, we observed huge effect sizes of >−2, indicating that words such as *healthy*, *supported*, *gain*, and *benefit* (from the LIWC-22 dictionary) were significantly less prevalent in the HiB-RC, suggesting that Redditors do not view hypersexuality as a rewarding behavior. Finally, the domain of *past focus* was significantly more prevalent in the HiB-RC, whereby manual analysis of posts suggests that Redditors were primarily recounting histories and past experiences of hypersexuality. The significantly lower presence of the *future focus* domain correlates with this finding, as well as signifying the impulsive nature of hypersexuality that has been documented in the literature [77,86].

### BERTopic Analysis

The clusters produced by BERTopic included 9 topics and 1 outlier class, and each topic was presented alongside a text excerpt from the most representative post (determined by BERTopic). Holistically, the model provided what we consider to be fairly distinct and identifiable topics, which is impressive considering the relatively small corpus and the niche domain of the dataset. Although topic modeling is not capable of capturing every nuance of the data, the model output provides a good starting point for understanding the data without needing to train a supervised model. The number of posts that were clustered into each topic by the model does not mean that these were the only posts that referenced a specific topic as some posts talked about more than one topic, and it is also likely that insightful data may have inadvertently been clustered into the outlier category. We can see that there was an increasing trend for all identified topics since 2017, which was especially pronounced for the topics of *sexuality* and *monogamy and polygamy* since 2020.

Evidence from the existing literature correlates with some of the topics identified by the automated model, including the onset of hypersexuality during an elevated mood [4,5,86], sexuality and sexual orientation [4,87], managing hypersexuality within a relationship [4,17], hypersexuality and medication [88-90], the role of child sexual abuse in hypersexuality [91-93], and vulnerability to sexual assault due to hypersexuality [27,77,82].

### The Utility of a Computational Linguistic Framework

Current evidence from lived experience underscores the severe and multifaceted consequences of hypersexuality. These include risks such as sexual assault, unplanned pregnancies, vulnerability to sexually transmitted infections, traumatic abortions, and significant disruptions in personal relationships [82]. Findings from a Bipolar Commission survey involving >1500 individuals reveal that 88% of respondents experienced hypersexual behaviors, highlighting the symptom’s prevalence and potential to impact thousands of people across the United Kingdom [27,94]. Over half of the participants reported experiencing ≥8 episodes of hypersexuality during their lifetime. Furthermore, 54% reported putting themselves in dangerous situations, 54% experienced relationship breakdowns, and 22% reported being raped during a period of hypersexuality. In total, 1 in 5 respondents attempted suicide due to hypersexual behavior or its consequences, aligning with previous findings that link hypersexuality in bipolar to increased suicidal ideation [95]. The data reveal a troubling gap in clinical practice, with 60% of respondents reporting that health care professionals had not addressed hypersexuality as part of their care [82]. This disconnect between the prevalence of hypersexuality and its clinical recognition underscores an urgent need for a more comprehensive understanding of hypersexual behaviors, particularly from the perspective of those with lived experience. The development of the HiB-RC and exploratory analysis using computational linguistic methods highlights the potential of this framework in advancing our understanding of hypersexuality as a symptom experienced by individuals with bipolar. The HiB-RC represents a significant resource for future research, enabling deeper exploration of the complex relationship between hypersexuality and bipolar to help bridge the gap between clinical knowledge and practice. The use of Reddit as a data source provides unique advantages, offering insights from real-time, user-generated narratives that are free from the constraints of predefined categories typically observed in self-report questionnaires or controlled laboratory settings [76]. This approach captures an authentic and dynamic perspective, reflecting the lived experiences of individuals as they occur. Future research using this dataset will use a corpus-assisted discourse analysis to explore key thematic concepts discussed by Reddit users and describe how these findings can inform and improve clinical practice for people with bipolar.

Additional avenues for future research could build on the exploratory nature of this study using alternative methodologies to verify the findings and deepen insights. For instance, ethnographic or participatory studies could provide a more immersive understanding, whereas large-scale qualitative studies using interviews could triangulate the results. Applying the same computational methods to clinical datasets would offer valuable cross-validation. Collecting more detailed demographic information, such as relationship status, could also shed light on how hypersexuality manifests across different life contexts, enriching our understanding of this complex symptom.

### Strengths and Limitations

This study offered a unique insight into the presentation of hypersexuality within a Reddit population who self-reported a professional diagnosis of bipolar. This is the first study to observe hypersexuality in such a population, and we endeavored to not only contribute to the literature on hypersexuality but also provide a rigorous and ethical framework for doing this. We used novel computational methods to identify salient patterns in the language used by Redditors, which signpost to common experiences shared by people who experience the symptom of hypersexuality. It is also important to consider the limitations of research conducted using social media data and predictive models, and these are outlined in this section.

First, as referenced in the Methods section, we relied on self-reported diagnoses of bipolar. As is the risk with any analysis conducted using social media data, we are assuming that the posts within our corpus are truthful. As described by Coppersmith et al [49], due to “the stigma often associated with mental illness,” it seems unlikely that Redditors would post about symptoms of a mental health condition that they do not have. We also tried to reduce false-positive reports of a bipolar diagnosis in the dataset by using pattern matching to capture self-reported diagnoses by Redditors.

Second, we also acknowledge limitations associated with demographic inference. The first limitation is that the gender inference model was restricted to the binary prediction of men and women as there is no tool currently available that predicts beyond these two genders, and this is a limitation of the demographic predictions. A tangential avenue for further research could involve the development of a multiclass predictive model to avoid binary classification. Future research that involves the collection of primary lived experience data (eg, through interviews) should also focus on inclusive data collection to encompass a broader set of gender identities. The second demographic limitation that we would like to address is that most of the inferred geolocations were based in America, and although the data that we report are consistent with existing literature on hypersexuality and bipolar, we cannot assume that these findings will be fully representative of international experiences. For example, Redditors worldwide are likely to be affected differently by varying health care provisions, which could have an impact on experiences with access to psychosocial support and medication costs.

Third, there are a number of limitations associated with using an unsupervised topic model, including the generation of a large number of outliers and a lack of objective evaluation metrics (which is consistent across topic-modeling methodologies). The interpretation of the topic models generated by BERTopic also still relies on human interpretation and domain knowledge, but BERTopic does provide an option to use an “auto” parameter in the setup of the model, which reduces the number of topics by merging similar clusters after the model has been trained to produce the “optimum” number of topics (as opposed to defining *k* number of topics in LDA). Finally, due to the stochastic nature of uniform manifold approximation and projection (the dimension reduction algorithm used by BERTopic), the resulting topics produced by the BERTopic model may differ when running the same code multiple times [29].

Finally, as we have acknowledged throughout this paper, we used a restrictive set of keywords to search for posts that contained references to hypersexuality, and therefore, the data presented in this paper are not definitively representative of all experiences and understandings of hypersexuality in bipolar across Reddit. Future research could use word embeddings on the HiB-RC to identify words and phrases that appear in a similar context to variants of the lemma *hypersexual* and then search for these words in the TABoRC to return a large corpus of posts that potentially describe hypersexuality. To avoid confusing hypersexuality with experiences of increased sex drive or discussion of nonnormophilic sexuality [16], these posts would need to be manually verified for inclusion, and strict coding guidelines would need to be developed.

### Conclusions

This paper has presented a novel methodology for generating a corpus of data related to experiences of hypersexuality in bipolar—inferring demographic information for these data—and 2 computational linguistic methods for exploratory analysis. We demonstrated that hypersexuality is an important symptom that is discussed by people living with bipolar, with significant associated factors suggested by the topic model, including the impact on relationships, discussion of medication, sexual assault, and correlation with an elevated mood. Our LIWC analysis demonstrated that posts describing hypersexuality were significantly more likely to include language that denoted mental illness and negative emotions, and we signposted to areas of further research that could be informative in guiding future clinical interventions. This study not only fills a critical gap by providing a dataset of experiences of hypersexuality within the context of bipolar but also highlights the potential of computational linguistic methods in mental health research. The findings underscore the importance of using innovative methodologies to bridge the gap between anecdotal experiences and empirical evidence, providing data that can help develop more informed and impactful psychosocial interventions in the future.

## Appendix

Multimedia Appendix 1 Annotation guidelines.

## Abbreviations

API: application programming interface
HiB-RC: Hypersexuality in Bipolar Reddit Corpus
LDA: latent Dirichlet allocation
LIWC: Linguistic Inquiry and Word Count
LIWC-22: 2022 version of Linguistic Inquiry and Word Count
TABoRC: Talking About Bipolar on Reddit Corpus

## Data Availability

A redacted version of both the Talking About Bipolar on Reddit Corpus and Hypersexuality in Bipolar Reddit Corpus are available from the UK Data Service.
