# Supplementary material for: Using Natural Language Processing Methods to Build the Hypersexuality in Bipolar Reddit Corpus: Infodemiology Study of Reddit
Source: JMIR Infodemiology. 2025 Mar 6;5:e65632. doi: 10.2196/65632 (PMC11926447; doi:10.2196/65632)
Supplement: Multimedia Appendix 1 [file infodemiology_v5i1e65632_app1.docx]

# Multimedia Appendix: Using Natural Language Processing methods to build the Hypersexuality in Bipolar Reddit Corpus (HiB-RC): an explorative study

## Appendix 1 – Annotation Guidelines for the HiB-RC

**Annotation Guidelines for Labelling Hypersexuality in Reddit Posts**

The aim of the annotation task is to create a dataset of users who identify as having experienced hypersexuality on Reddit. The posts of these users will be used for further linguistic analysis related to sexual risk-taking behaviours.

There are three possible labels to annotate each post, but each post should only receive one label.

- **Hypersexuality**: For posts where the user mentions that they have experienced hypersexuality (either presently or in the past). This may be a brief mention as part of a list of other behaviours but must refer to the author and not generally to a list of symptoms which relate to bipolar e.g. ‘During hypomania I experience hypersexuality, racing thoughts and I spend too much money’.
- **Negative**: For posts where hypersexuality is mentioned but not specifically in relation to the author, or where the post doesn’t contain any reference to hypersexuality e.g. ‘Common symptoms during hypomania can include hypersexuality, racing thoughts and spending too much money’. These posts will either be used to create a binary classifier for hypersexuality, or they may be
- **Ignore**: For posts which are too difficult to understand, or where they are written in a language other than English. These posts will be removed to prevent noise.

**Annotating in Excel**

- The post content is saved in the ‘text’ field
- Labels should be selected from the dropdown menu in the ‘label’ field
- Note: you might see some Unicode symbols e.g. ‚Ä¶, these can be ignored

**Annotating in Doccano**

- See the documentation here: <https://doccano.github.io/doccano/install_and_upgrade_doccano/#install-with-docker> for installing Doccano through Docker (you will need to include your email address and create a password as part of the installation but you only need to do this once)
- Once Doccano has been installed, run it either directly from Docker desktop app or through the command line
- The webserver will prompt you to login to Doccano using the credentials you provided at installation (email address and password)
- Once you’ve created an account I can add you to the project
- Click ‘start annotation’ and start selecting labels for the posts – it will show as 0% progress (but will show the number of posts you’ve added a label for) until we approve the labels during resolution stage
